# Supplementary material for: Longitudinal cardiorespiratory wearable sleep staging in the home
Source: Front Neurosci. 2026 Feb 17;20:1693860. doi: 10.3389/fnins.2026.1693860 (PMC12953379; doi:10.3389/fnins.2026.1693860)
Supplement: Supplementary file 1 [file Data_Sheet_1.pdf]

Supplementary Material

1 EXTRACTING A RESPIRATORY SIGNAL FROM THE ACCELEROMETER

The ACC-RESP signal was extracted using the following process:

1. Low-pass filter the three-axis accelerometer signal using a 3rd order Butterworth filter with a cut-off frequency of 3.5 Hz.
2. Linearly re-sample the filtered accelerometer signal down to 16 Hz, giving the three-axis accelerometer signal $\vec{a}$.
3. Compute the gravity vector at the sample point n as the recursive, rolling average of the three-axis accelerometer signal using equation S1.

$$\vec{g}[n] = \alpha_h \vec{a}[n-1] + (1-\alpha_h)\vec{g}[n-1] \tag{S1}$$

   Where $\vec{g}$ is the gravity vector (which is initialised with the value [0 0 1]) and α_h is a coefficient described in Table S1. The gravity vector is used to determine whether movement is occurring using equation S2.

$$m[n] = \|\vec{a}[n] - \vec{g}[n]\| > 0.06\|\vec{g}[n]\| \tag{S2}$$

   Where $m[n]$ is a movement flag, which is applied for the subsequent 2.5 seconds (15 samples) when movement occurs and affects the coefficients described in Table S1.

4. For each of the three accelerometer axes, remove the DC component, thereby extracting the respiratory component of the signal, by subtracting the 5-second (80-sample) moving mean, giving the three-axis respiratory signal $\vec{h}$. This is in contrast to the method described in Schipper et al. (2021), in which the gravity vector is used to determine the direction of respiratory motion, giving a two-axis signal.
5. Recursively normalise the respiratory signal $\vec{h}$ using equation S3.

$$\vec{u}[n] = \frac{\vec{h}[n]}{p[n]} \tag{S3}$$

   where $\vec{u}$ is the normalised respiratory signal and p is a rolling, recursive norm computed using equation S4.

$$p[n] = \alpha_n \|\vec{h}[n]\| + (1-\alpha_n)p[n-1] \tag{S4}$$

   where α_n is a coefficient described in Table S1 and p is initialised by taking the mean norm of the first 10 samples of $\vec{h}$

6. Low-pass filter the normalised respiratory signal $\vec{u}$ using a 3rd order Butterworth filter with a cut-off frequency of 0.8 Hz, giving the output signal $\vec{v}$.
7. Perform recursive PCA on the three-axis respiratory signal $\vec{v}$ (as opposed to the two-axis signal in Schipper et al. (2021)) using equation S5.

$$\hat{r}[n] = \vec{w}[n] \cdot \vec{v}[n] \tag{S5}$$

where $\hat{r}[n]$ is the accelerometer-derived respiratory signal (ACC-RESP), and the direction of principal component $\vec{w}$ is approximated recursively using equation S6.

$$\vec{w}[n+1] = \eta\hat{r}[n]\vec{v}[n] + (1 - \eta\hat{r}[n]^2)\vec{w}[n] \quad \text{(S6)}$$

where η is a learning rate described in Table S1, and $\vec{w}$ is arbitrarily initialised as [1 0 0].

8. Adjust the sign of the ACC-RESP signal to ensure that inspiration and expiration have consistent directions (positive and negative, respectively). This is achieved using the skewness s, which is recursively approximated using equation S7.

$$s[n] = \alpha_s\hat{r}[n]^3 + (1 - \alpha_s)s[n-1] \quad \text{(S7)}$$

where α_s is a coefficient described in Table S1. If the skewness falls below a threshold of 5e-3 at a sample point n, the direction of inspiration and expiration are assumed to be incorrect, and the skewness $s[n]$ and projection axis $\vec{w}[n]$ at that sample point are inverted.

An example segment of night time chest-patch accelerometer data with the associated accelerometer-derived respiratory waveform is shown in fig. S1.

Table S1. Movement-affected coefficients for extracting a respiratory waveform from three-axis accelerometer, as established in Schipper et al. (2021). The greater ‘Body Movement’ values allow the recursive method to adapt more rapidly after a potential change in orientation by a participant.

Parameter	Normal Value	Body Movement Value
α_h	0.03	0.30
α_n	0.01	0.10
α_s	7e-5	7e-4
η	8e-4	8e-3

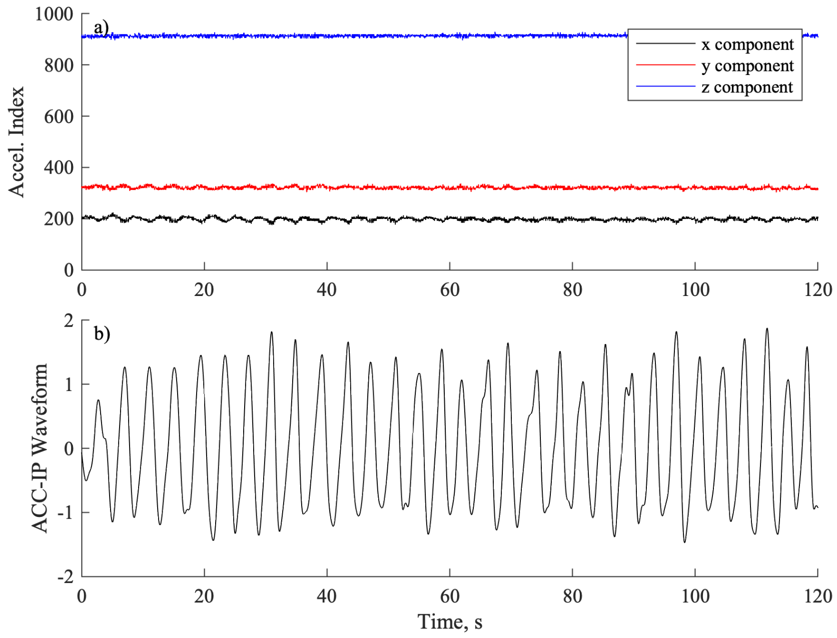

Figure S1. Example 120 second segment of overnight waveforms for: a) chest-patch derived 3-axis accelerometry at 125 Hz; b) accelerometer-derived respiratory waveform (ACC-RESP).

REFERENCES

Schipper F, van Sloun RJ, Grassi A, Derkx R, Overeem S, Fonseca P. Estimation of respiratory rate and effort from a chest-worn accelerometer using constrained and recursive principal component analysis. *Physiological Measurement* **42** (2021) 045004.
